# In-Person or Online? Exploring Student Pharmacists’ Perceived Change in Interprofessional Skills between Two Delivery Formats

**DOI:** 10.3390/pharmacy11020055

**Published:** 2023-03-14

**Authors:** Jared Van Hooser, Anthony W. Olson

**Affiliations:** 1 Department of Pharmacy Practice and Pharmaceutical Sciences, University of Minnesota College of Pharmacy, Duluth, MN 55812, USA; 2 Essentia Institute of Rural Health, Duluth, MN 55805, USA

**Keywords:** pharmacy education, interprofessional education (IPE), simulations, remote learning, experiential learning, COVID-19, simulation, standardized patients

## Abstract

The COVID-19 pandemic drastically changed how education is delivered. Many academic programs quickly transformed their curriculum to online distance learning. This rapid transition may have compromised the rigor and fidelity of these activities. The Interprofessional Standardized Patient Experience (ISPE) is an interprofessional education activity (IPE) involving a team of students from six different healthcare professions that switched to an online delivery format. This manuscript compares pharmacy students’ perceived change in interprofessional skills between the two formats. Following the ISPE, second-year pharmacy students completed the revised Interprofessional Collaborative Competencies Attainment Survey (ICCAS-R). The ICCAS-R assesses the change in interprofessional collaboration-related competencies in healthcare students before and after IPE training using a retrospective pre–post approach. For each ICCAS-R item and each delivery format (44 in-person and 51 online), paired Student’s t-test on pre- and post-ISPE scores, and Cohen’s d were calculated. Every item of the ICCAS-R was significant (*p* < 0.001), regardless of delivery format. Nearly all ICCAS-R items had a large effect size, and the remaining items had a medium effect size. The amount of change pre- and post-ISPE for each ICCAS-R item was calculated. Student’s t-test was used to compare the magnitude of change in interprofessional skills between the two delivery formats. Only one difference was noted between the two delivery formats—ICCAS-R item 16, which measured actively listening to interprofessional team members (*p* = 0.0321). When switching to an online format, the high-fidelity dimension of the ISPE was retained. The ISPE is an effective IPE activity at increasing pharmacy students’ self-perceived interprofessional collaboration skills regardless of delivery format. Even though students reported the ISPE increased their ability to actively listen to the perspectives of interprofessional team members in both formats, the magnitude of the benefit was more profound in the in-person group.

## 1. Introduction

In the Spring of 2020, the COVID-19 pandemic forced many academic programs to transform their curriculum from primarily in-person to exclusively online distance learning. Activities designed and optimized for in-person, synchronous content delivery (i.e., singular time and place to all students) in courses were quickly adapted for synchronous, online delivery to each individual student’s remote location. This raised the question: were students getting “the same” quality of education and achieving the same outcomes?

A growing body of evidence suggests that online, asynchronous educational approaches, (e.g., flipped classroom model) result in either no difference or even favorable educational outcomes and satisfaction when compared to traditional, in-person instruction [1,2]. Similarly, a recent meta-analysis resulted in no significant differences in effectiveness between health science students in courses with solely in-person instruction and those with synchronous distance education models where there were at least two separate large groups of students in separate locations (i.e., satellite or multiple campuses connected virtually in real-time) [3]. However, there is limited knowledge on the effectiveness of synchronous, online activities in health professional education, with all participants located in separate locations. The conditions presented by the COVID-19 pandemic presented an opportunity to conduct a natural experiment addressing this gap through the participation of student pharmacists in an education activity known as the Interprofessional Standardized Patient Experience (ISPE).

The ISPE is an interprofessional education activity lasting 1–2 h involving a team of students from six different healthcare professions: nursing, medicine, pharmacy, social work, physical therapy, and occupational therapy [4,5]. Participating student pharmacists found their own means of transportation off-campus to a nearby private college with a nursing program in the Duluth, MN area where the activity was conducted. Prior to March 2020, students from each profession separately interviewed the same standardized patient in-person with a case containing subjective data unique to each respective profession developed by an interprofessional faculty team. When students were not interviewing the standardized patient in this simulated out-patient setting, they observed their team members doing so through a one-way mirror. This allowed for each successive team member to build upon the information collected by the previous students in their own interviews with the patient. After all subjective interviews were completed, a faculty member led a small group discussion with each team reflecting on the interview, interprofessional collaboration, and the benefits and barriers of interprofessional practice. Student pharmacists then completed an electronic post-ISPE survey to self-assess their change in interprofessional collaboration skills. In March 2020, the ISPE transitioned to a synchronous online delivery format using the videotelephony software program Zoom [6], but otherwise mirroring the in-person process.

The objective of this study was to explore differences between the effectiveness of ISPE when delivered synchronously in-person and synchronously online by comparing student pharmacists’ self-perceived changes in interprofessional collaboration skills and the experience for each delivery method.

## 2. Materials and Methods

### 2.1. Data Collection

Data for this retrospective, ecological study were obtained via online surveys administered in a learning management system to 95 s-year student pharmacists enrolled in the Community Teacher Experience I and II courses (PHAR 7330/40) at the University of Minnesota, College of Pharmacy, and who completed the ISPE. Surveys were administered between September 2019 and April 2021 via the course’s webpage, which was hosted by the learning management system Canvas [7]. Of the 96 enrolled students, 95 completed the ISPE. Forty-four participated in the synchronous, in-person version of the ISPE between September 2019 and March 2020. The remaining 51 students completed the synchronous, online version between March 2020 and April 2021 (i.e., after COVID-related closures). All students were required to complete the ISPE and accompanying surveys in order to pass the PHAR 7330/40 courses. In a single survey sent after completing the ISPE, students were asked to self-assess their interprofessional skills both before and after the ISPE (i.e., retrospective pre–post approach) and answer questions related to the activity.

### 2.2. Study Variables

The independent variable of this study was whether students completed the ISPE synchronously in-person or synchronously online. The dependent variables consisted of 20 items comprising revised Interprofessional Collaborative Competencies Attainment Survey (ICCAS-R) [8,9] (scale: 1 = poor→5 = excellent), as well as three unvalidated forced-choice questions and a free text response question evaluating the student’s experience participating in the ISPE. The ICCAS-R assesses the change in interprofessional collaboration-related competencies in healthcare students on a five-point scale from poor to excellent [8,9]. The survey’s three unvalidated items asked students to respond to the following prompts and questions: [1] “Compared to the time before the learning activities, would you say your ability to collaborate inter-professionally is” (scale: 1 = much worse now→5 = much better now); [2] “How well did the activity meet the learning objectives?” (1 = none, 2 = few, 3 = most, 4 = all); and [3] “Was the activity fun?” (yes, no). The final item on the survey asked for an open-ended response to the statement, “Please add any additional comments you have about this activity”.

### 2.3. Data Analysis

For each ICCAS-R item and each delivery format, a pre- and post-ISPE mean and paired Student’s t-test was calculated. Cohen’s d effect size, which expresses the size of an effect as a number of standard deviations, was also calculated for each ICCAS-R item. Cohen’s d values 0.2 to 0.5 were considered a small effect size, greater than 0.5 to 0.8 represented a medium effect size, and greater than 0.8 a large effect size [10].

A pre- and post-ISPE magnitude of change value for each ICCAS-R item was calculated for each delivery format by subtracting pre-IPSE values from paired post-ISPE values. The magnitude for each ICCAS-R item between the two delivery groups was compared with Student’s t-test.

A Mann–Whitney U test was used to compare the responses to the first two forced-choice questions related to the student’s experience taking the ISPE between delivery formats. Responses to “Was the activity was fun?” were compared with Fisher’s exact test. Finally, free text responses were reviewed for any responses relating to the delivery format. Statistical significance was specified at *p* value < 0.05, and JMP Pro 16 (SAS Institute Inc. Cary, NC) was used for analysis [11].

The Benjamini–Hochberg procedure was used to account for false discoveries of statistical significance at a threshold of 5% given the high number of tests conducted [12]. Accordingly, all calculated *p*-values were ordered from smallest to largest and input into the Benjamini–Hochberg formula (i/m)*Q = B, whereas:

- i = *p* value rank order (i.e., #1-#63)
- m = 63 (i.e., total number of tests)
- Q = 0.05 (i.e., false discovery rate)
- B = Benjamini–Hochberg critical value

Following this procedure, all tests where a test’s *p* value is smaller than the Benjamini–Hochberg critical value retain statistical significance.

## 3. Results

All 95 students who participated in the ISPE completed the online survey. Table 1 displays the mean pre- and post-ISPE responses for both comparison groups across the 20 items of the ICCAS-R. Every item of the ICCAS-R was significant after applying the Benjamini–Hochberg procedure (*p* < 0.001), regardless of delivery format. Nearly all ICCAS-R items had a large effect size (Cohen’s d ≥ 0.8), and the remaining items had a medium effect size (Cohen’s d ≥ 0.5).

Only one difference after the application of the Benjamini–Hochberg procedure was noted in the magnitude of change values between the two delivery formats: ICCAS-R item 16, which measured actively listening to interprofessional team members (*p* = 0.0321; B = 0.0325). All other magnitude of change value comparisons were non-significant.

There was no difference between students’ reported increase in their ability to collaborate interprofessionally when the ISPE was delivered in-person (mean 4.2) compared to online delivery (mean 4.1) (*p* = 0.5005). No difference was found between delivery formats in how well the ISPE met the learning objectives (in-person mean 3.4; online mean 3.4; *p* = 0.7189).

Of the in-person cohort, 43/44 (98%) reported the activity was fun, and 49/51 (96%) reported the activity was fun from the online cohort (*p* = 0.8495). All responses specifically related to the delivery format to the optional free text question are below (edited to remove identifying information).

- In-person
  ○ Finding the room at the college.
- Online
  ○ Unfortunately, our session was over Zoom and our mock patient was cutting in and out a couple times throughout our interview.
  ○ I think it was great. I think it worked well via Zoom as well.
  ○ I thought doing this activity over Zoom was just as effective as if it had been live! I really enjoyed the process.
  ○ It was fun to see how each profession went about the case. Each profession had a certain area that they tried to focus on. I am sad that it had to be over Zoom!
  ○ The least favorite part of this activity was having to do this experience over Zoom. It would be better in-person.

## 4. Discussion

The totality of the findings suggests that the ISPE is an effective interprofessional education activity for increasing pharmacy students’ self-perceived interprofessional collaboration skills, whether delivered in-person or online. Between the delivery methods, the sole statistically significant difference was students’ ability to actively listen to the perspectives of interprofessional team members. The magnitude of benefit was greater in the in-person group, but both ISPE delivery formats produced increases on this item. Thus, reviewing best practices and strategies for active listening in online environments prior to an online ISPE’s delivery may help mitigate this difference. Another factor that may even this difference is additional exposure and practice students may have with virtual interactive sessions in a post-COVID environment.

To further support that the effectiveness of the ISPE was retained in the asynchronous, online format, no difference was found in students pharmacists’ responses in the ISPE’s capacity to increase their ability to collaborate interprofessionally and meet the learning objectives. Additionally, nearly all students (92/95) reported having fun during the activity. Though the items producing these findings were unvalidated, they align well with the ICCAS-R results indicating little to no meaningful difference in the student experience of the ISPE. The ISPE continuing to be fun when conducted synchronously online is particularly notable, given correlations between fun, learning, and memory [13].

For the free text response item, five of the six usable responses about the ISPE delivery format came from students who completed the synchronous, online ISPE. This distribution is likely attributable to the possibility of an online ISPE and the COVID-related conditions leading to its implementation not being anticipated by students completing the in-person activity. The only response from a student who completed the in-person ISPE cited the difficulty they experienced finding the ISPE’s location, which was held off-campus at a site unfamiliar to student pharmacists. The remaining five responses from students participating in the online ISPE provided mixed reviews about their experience and medium preferences. Two responses were positive in valence, indicating an effective, virtual experience (e.g., “Zoom was just as effective as if it had been live! I enjoyed the process”), whereas the remaining three responses were negative in valence, citing either technical difficulties (e.g., “mock patient was cutting in and out (over Zoom)”) or a preference for being in-person for the activity (e.g., “(the activity) would be better in-person).

The overarching results in this study indicate little to no difference in the effectiveness of this activity; the responses from the free text item, in particular, suggest this may not correlate with satisfaction. Though the limited number and detail of the free text responses prevent this from being a strong inference, the idea is supported by findings from other studies comparing online-synchronous, online-asynchronous, and in-person instructional approaches [14,15]. Some students may prefer online experiences for synchronous activities because of the flexibility it can provide (e.g., being able to participate from a physical location of their choosing, saving time and money, ability to participate even if self-isolating) [16,17]. Other students may prefer in-person synchronous activities due to unfamiliarity and discomfort with using virtual technology, unreliable Internet connections, and difficulties staying focused while on a computer [15]. The diversity of student preferences, needs, and abilities in each learning environment suggests that a blended approach combining elements online-synchronous, online-asynchronous, and in-person may be optimal. Furthermore, different types of educational activities and exercises may be more conducive to one approach than another [15].

Future research with an expanded sample size, a prospective study design, and controls for gender, age, race/ethnicity, and socioeconomic status is needed to assess the validity and reproducibility of these findings. Though the results with student pharmacists are promising, the impact of the ISPE delivery format with the participating non-pharmacist professions, nursing, medicine, social work, physical therapy, and occupational therapy should be evaluated to assess for any differences. Additionally, repeated assessment of the online delivery format ISPE will provide insight into student expectations and how they might change over time, answering the question: did students have lower expectations for online activities at the start COVID-19 pandemic when activities were quickly moved online compared to several years after the start of the pandemic? In other words, are responses falsely elevated at the start of the pandemic as students were giving facilitators grace as facilitators and standardized patients navigated new technology, logistics, and workflows? It is unclear if students further along in the PHAR 7330/40 courses and, thus, further along in the curriculum overall had an advantage over students who completed the ISPE earlier. The ISPE focuses on collecting subjective information unique to each respective profession, observing other professions collect information, and an interprofessional discussion instead of determining appropriate clinical solutions and plans. Any advantage is unlikely, as the ISPE is centered on the Interprofessional Education Collaborative’s Core Competencies rather than clinical content [18]. 

This exploratory study indicates that the ISPE is an effective interprofessional education activity for increasing pharmacy students’ self-perceived interprofessional collaboration skills, regardless of delivery format. Other interprofessional education activities should test effectiveness, as their activities were likely moved online due to COVID-19. Interprofessional education activity leaders will need to determine if the advantages of online delivery (e.g., no commuting or transition time for students and faculty, no room reservations/physical space logistics, etc.) outweigh any differences in activity effectiveness, if found, and merit keeping interprofessional education activities online even if the in-person delivery is available.

## Figures and Tables

**Table 1:** Average ICCAS-R responses pre- and post-ISPE for in-person and online delivery formats with respective effect size (*N* = 95).

Construct	ICCAS-R Item Before/After Participating in the Learning Activities, I Was Able to:	In-Person (*N* = 44)				Online (*N* = 51)			
		Pre- ISPE Mean	Post-ISPE Mean	Pre- Post *p* Value	Cohen’s d	Pre- ISPE Mean	Post-ISPE Mean	Pre- Post *p* Value	Cohen’s d
Communication	1. Promote effective communication among members of an IP team	3.09	3.75	<0.0001	1.28	3.16	3.78	<0.0001	1.19
	2. Actively listen to IP team members’ ideas and concerns	3.61	4.18	<0.0001	1.04	3.49	4.14	<0.0001	1.28
	3. Express my ideas and concerns without being judgmental	3.57	4.11	<0.0001	1.00	3.59	4.14	<0.0001	1.05
	4. Provide constructive feedback to IP team members	2.82	3.23	<0.0001	0.69	2.71	3.29	<0.0001	0.96
	5. Express my ideas and concerns in a clear, concise manner	3.07	3.57	<0.0001	0.85	3.16	3.69	<0.0001	0.91
Collaboration	6. Seek out IP team members to address issues	2.91	3.55	<0.0001	0.93	2.96	3.71	<0.0001	1.29
	7. Work effectively with IP team members to enhance care	3.18	3.98	<0.0001	1.55	3.16	3.92	<0.0001	1.37
	8. Learn with, from, and about IP team members to enhance care	3.07	4.02	<0.0001	1.74	3.24	4.04	<0.0001	1.56
Roles and responsibilities	9. Identify and describe my abilities and contributions to the IP team	3.02	3.86	<0.0001	1.38	3.27	3.94	<0.0001	1.32
	10. Be accountable for my contributions to the IP team	3.36	3.91	<0.0001	0.94	3.55	4.00	<0.0001	0.76
	11. Understand the abilities and contributions of IP team members	2.95	4.00	<0.0001	1.80	2.94	3.98	<0.0001	1.65
	12. Recognize how others’ skills and knowledge complement and overlap with my own	2.98	4.18	<0.0001	2.20	2.90	4.00	<0.0001	1.96
Patient-centered care	13. Use an IP team approach with the patient to assess the health situation	2.95	3.84	<0.0001	1.65	3.06	3.94	<0.0001	1.59
	14. Use an IP team approach with the patient to provide whole person care	2.95	3.95	<0.0001	2.13	3.06	3.98	<0.0001	1.58
	15. Include the patient/family in decision-making	2.91	3.55	<0.0001	0.95	3.16	3.63	<0.0001	0.76
Conflict management, team functioning	16. Actively listen to the perspectives of IP team members	3.39	4.20	<0.0001	1.41	3.59	4.10	<0.0001	1.05
	17. Take into account the ideas of IP team members	3.32	4.09	<0.0001	1.36	3.45	4.18	<0.0001	1.37
	18. Address team conflict in a respectful manner	3.25	3.61	<0.001	0.58	3.16	3.49	<0.001	0.55
	19. Develop an effective care plan with IP team members	3.16	3.80	<0.0001	1.12	3.00	3.55	<0.0001	0.89
	20. Negotiate responsibilities within overlapping scopes of practice	3.09	3.77	<0.0001	1.30	2.94	3.59	<0.0001	1.15

Abbreviations: IP = interprofessional; ISPE = Interprofessional Standardized Patient Experience. Cohen’s d values effect size: 0.2 to 0.5 small, >0.5 to 0.8 medium, and >0.8 large.

## Data Availability

Not applicable.
